# Disciform Keratitis That Improves With Poly(Adenosine Diphosphate-Ribose) Polymerase Inhibitor Treatment for Ovarian Cancer but Recurs Upon Discontinuation of Treatment

**DOI:** 10.7759/cureus.116202

**Published:** 2026-09-14

**Authors:** Atsuki Fukushima, Shintaro Kohno, Hitoshi Tabuchi

**Affiliations:** 1 Ophthalmology, Tsukazaki Hospital, Himeji, JPN; 2 Ophthalmology, Hiroshima University, Hiroshima, JPN

**Keywords:** cancer, disciform keratitis, herpes virus, immune response, immunocompromised, infection

## Abstract

The patient was a 73-year-old woman. She underwent surgery for ovarian cancer but needed chemotherapy because tumor cells were observed at the resection margin. She was administered niraparib tosilate hydrate, a poly(adenosine diphosphate-ribose) polymerase (PARP) inhibitor, for five years; however, she developed severe malaise that made daily life difficult, rendering continued treatment impossible. Consequently, the PARP inhibitor was discontinued, and her physical condition improved. One year after discontinuation, she developed ocular herpes zoster, followed by disciform keratitis, leading to her presentation at our hospital. Examination of the right eye revealed disciform corneal edema and opacity, resulting in a diagnosis of disciform keratitis. Varicella-zoster virus (VZV) DNA was detected via PCR analysis of the aqueous humor; treatment with oral valacyclovir and topical betamethasone was initiated. Subsequently, the disciform keratitis resolved with continued topical betamethasone therapy. However, ovarian cancer recurrence was observed, and another PARP inhibitor, olaparib, was prescribed. She again experienced malaise, necessitating the discontinuation of the PARP inhibitor. One month after discontinuation, a recurrence of disciform keratitis was observed. Frequent topical betamethasone administration was initiated, resulting in the resolution of the disciform keratitis. These findings suggest the possibility that in patients with cancer who are immunocompromised due to treatment with PARP inhibitors, an immune response against VZV does not occur; however, when immune function recovers upon discontinuation of the PARP inhibitor, the immune response against VZV is reactivated, resulting in the recurrence of disciform keratitis. Thus, when managing patients with cancer, it is essential to monitor fluctuations in immune response while addressing ocular infections and inflammatory diseases.

## Introduction

A condition in which the cornea becomes swollen in a disc-shaped pattern and cloudy is called disciform keratitis. Initially, disciform keratitis was known as "Arlt's abscessus siccus." In 1901, Fuchs renamed the condition "disciform keratitis" [1]. Disciform keratitis causes visual impairment due to corneal edema, opacity, and anterior chamber inflammation; furthermore, it can lead to a loss of corneal endothelial cells and result in bullous keratopathy [2]. Although the etiology of disciform keratitis was initially unclear, reports subsequently identified herpesviruses as a primary cause [3]. Regarding the pathogenesis, as the concept of immunology gained academic recognition, it was demonstrated that the condition involves not merely viral infection but also the host's biological response, specifically an immune response to viral antigens, which plays a crucial role [4]. It is now understood that the pathophysiology of disciform keratitis involves corneal stromal infiltration (predominantly by mononuclear leukocytes such as lymphocytes) and keratic precipitates, accompanied by disciform stromal edema and microcystic edema localized to the affected area [5]. Since these inflammatory cells infiltrate the eye locally via the systemic circulation, systemic immune status may significantly influence the onset of disciform keratitis. Recent reports indicate that, in addition to herpesviruses, other pathogens such as *Acanthamoeba* [6] and microsporidia [7] can also induce disciform keratitis.

As noted above, while disciform keratitis may be triggered by an infectious process, the immune response plays a primary role in the development of the clinical presentation. While immunosuppression typically exacerbates rather than cures viral infections, it can serve to suppress inflammation in cases where the immune response to viral antigens triggers an inflammatory reaction. This report discusses a case in a patient with cancer in which the administration of anticancer drugs influenced the resolution and recurrence of disciform keratitis.

## Case presentation

The patient was a 73-year-old woman. She underwent surgery for ovarian cancer but continued chemotherapy because tumor cells were observed at the resection margin. She was treated with the anticancer drug niraparib tosilate hydrate, a poly(adenosine diphosphate-ribose) polymerase (PARP) inhibitor, for five years; however, she developed severe malaise that made daily activities difficult (Common Terminology Criteria for Adverse Events (CTCAE): grade 3), rendering continued treatment impossible. Consequently, the PARP inhibitor, which had been administered for five years, was discontinued, and her physical condition improved. One year after discontinuation, she noticed blurred vision with ocular pain and hyperemia. A few days later, she was diagnosed with ocular herpes zoster, followed by disciform keratitis, leading her to visit our hospital. At the initial examination, her best-corrected visual acuity (BCVA) was 1.2 in both eyes, and intraocular pressure was 12 mmHg in the right eye and 11 mmHg in the left eye. Disciform corneal edema and opacity, together with keratic precipitates and anterior chamber cells, were observed in the right eye (Figure 1), and a diagnosis of disciform keratitis was made.

**Figure 1:**
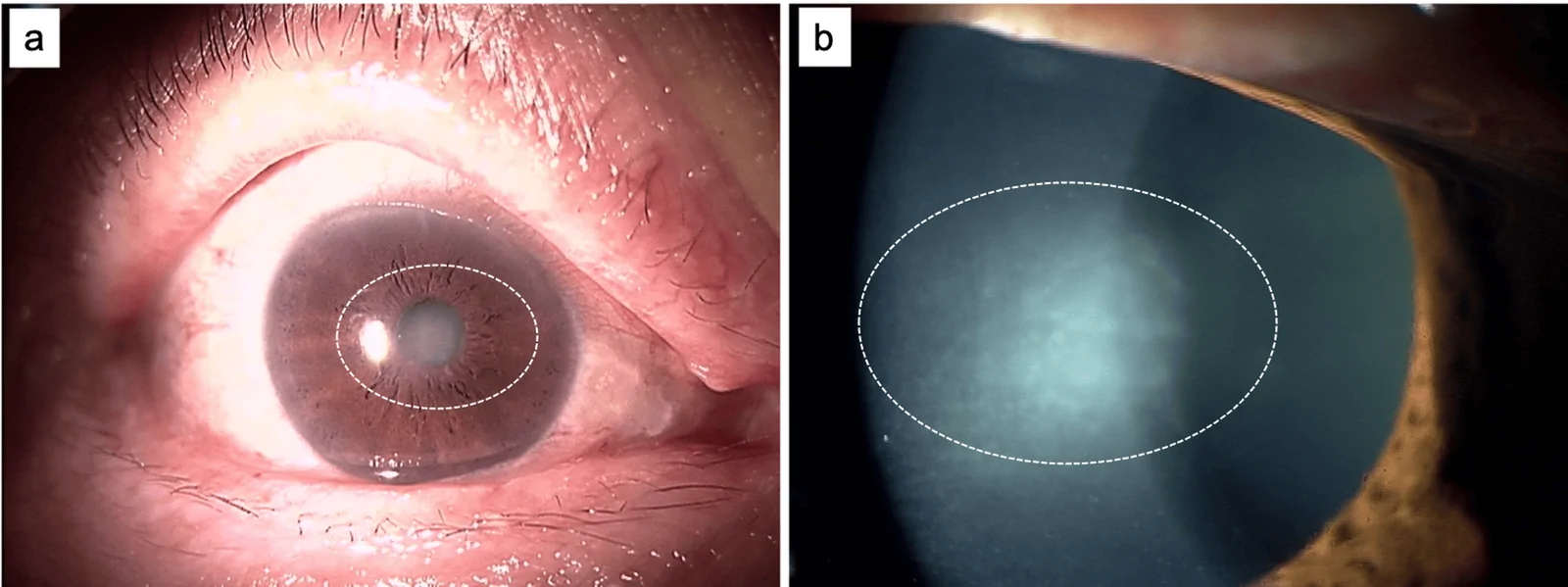
Slit-lamp photographs at the initial visit. (a) Low magnification; (b) high magnification. Disciform keratitis is observed in the central cornea (dotted circle).

Varicella-zoster virus (VZV) DNA was detected via PCR analysis of the aqueous humor; the patient was treated with oral valacyclovir for seven days and topical betamethasone, resulting in improvement.

**Table 1:** PCR results of aqueous humor samples.

Type of pathogen	PCR result
Herpes simplex virus type 1	Negative
Varicella-zoster virus	Positive
Herpes simplex virus type 2	Negative
Human herpesvirus 6	Negative
Epstein-Barr virus	Negative
Cytomegalovirus	Negative
Human T-cell leukemia virus type 1	Negative
Treponema pallidum	Negative
Toxoplasma gondii	Negative

Subsequently, the disciform keratitis resolved with continued topical betamethasone therapy (Figure 2).

**Figure 2:**
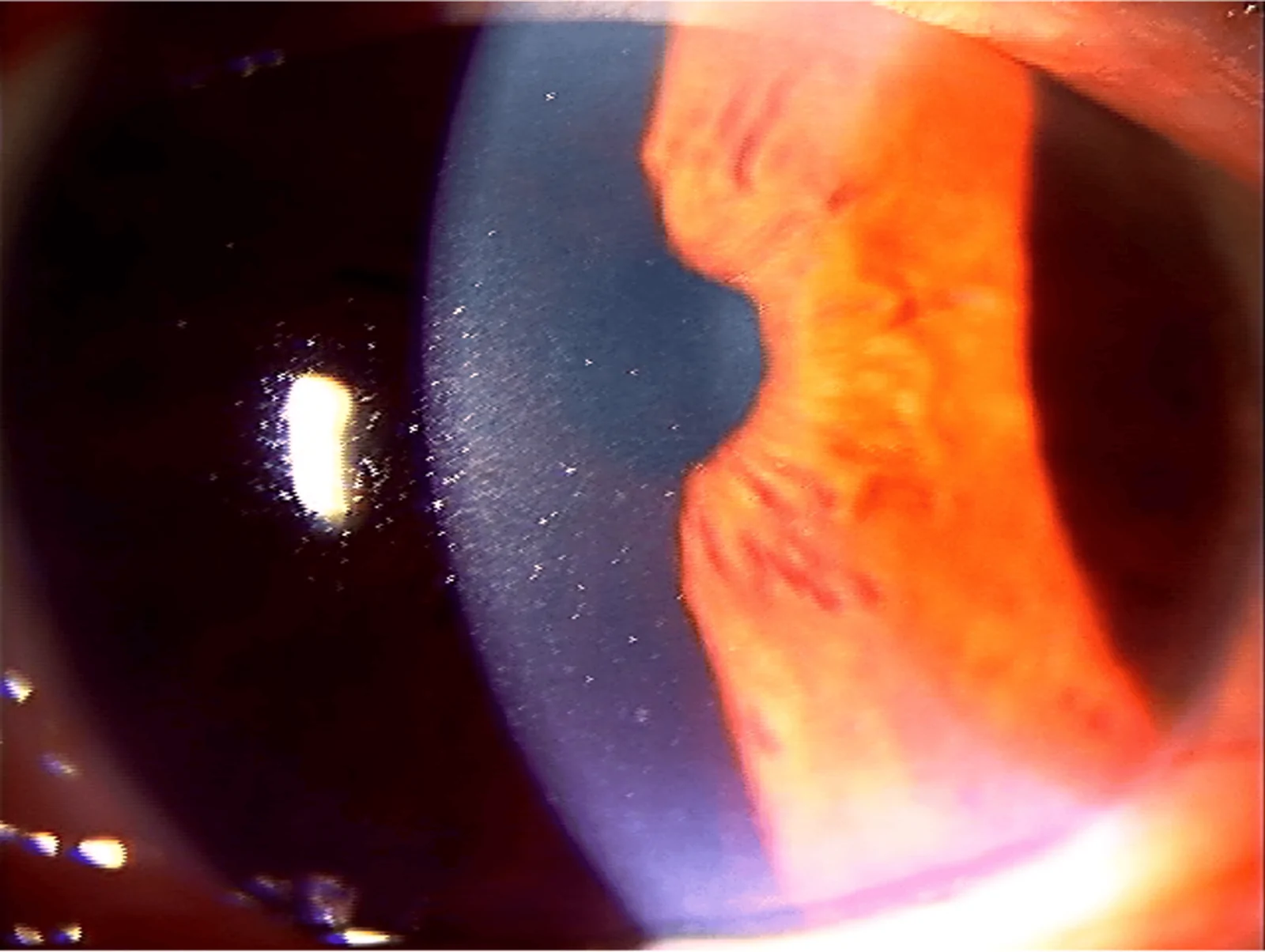
Slit-lamp photograph two months after the initial visit. Disciform keratitis had resolved.

Topical betamethasone therapy was completed six months after it began. Following a recurrence of ovarian cancer, the patient was prescribed olaparib, a PARP inhibitor. However, the patient again experienced physical deterioration (CTCAE: grade 3), leading to the discontinuation of the PARP inhibitor. One month after discontinuation, a recurrence of disciform keratitis was observed using the retroillumination method (Figure 3) because the lesion was mild.

**Figure 3:**
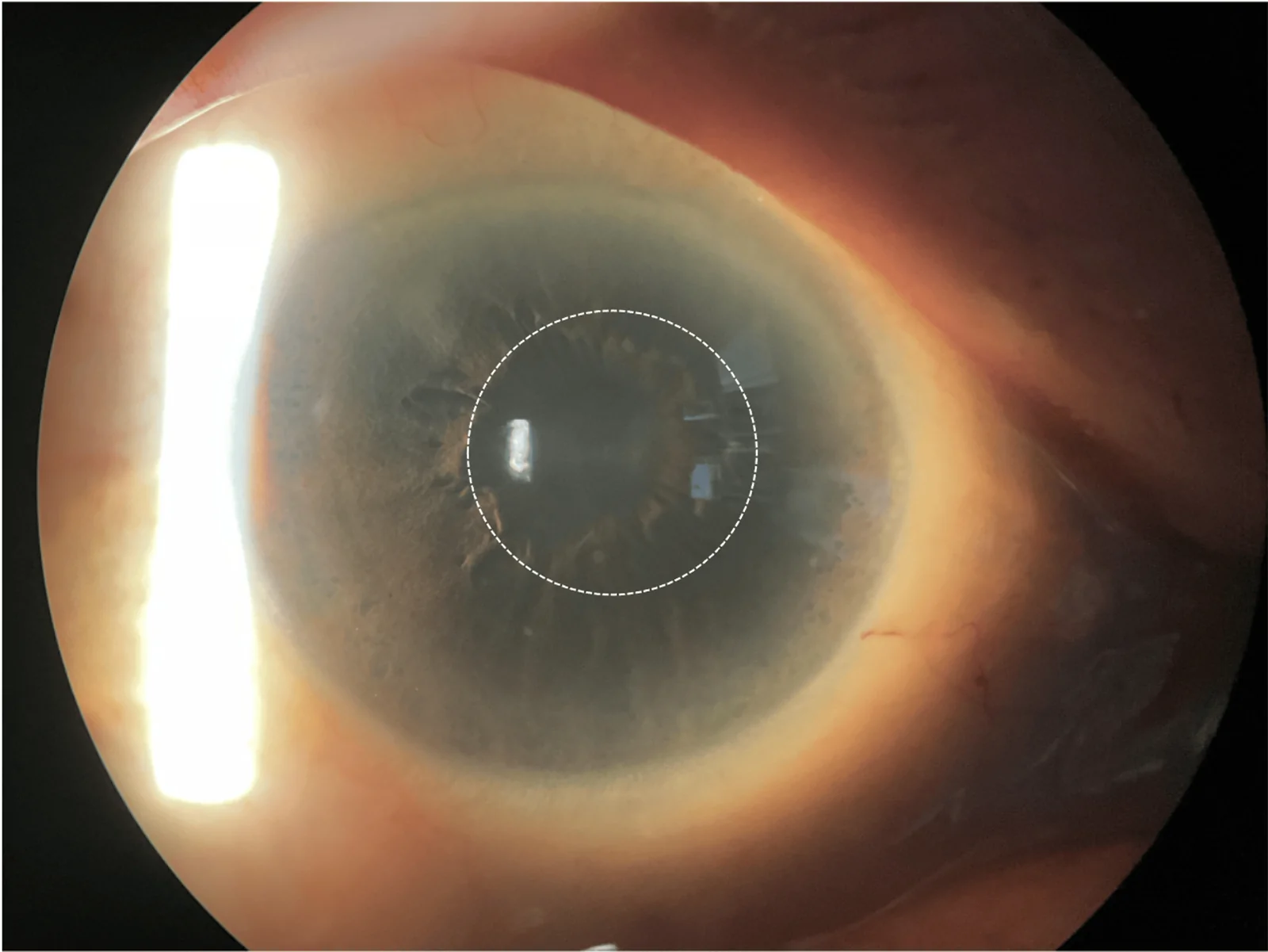
Slit-lamp photograph one month after discontinuation of the PARP inhibitor olaparib. Retroillumination image. Recurrence of disciform keratitis is observed (dotted circle). PARP: Poly(adenosine diphosphate-ribose) polymerase.

Consequently, frequent topical betamethasone administration was initiated; the disciform keratitis improved, and there has been no recurrence for three months (Figure 4).

**Figure 4:**
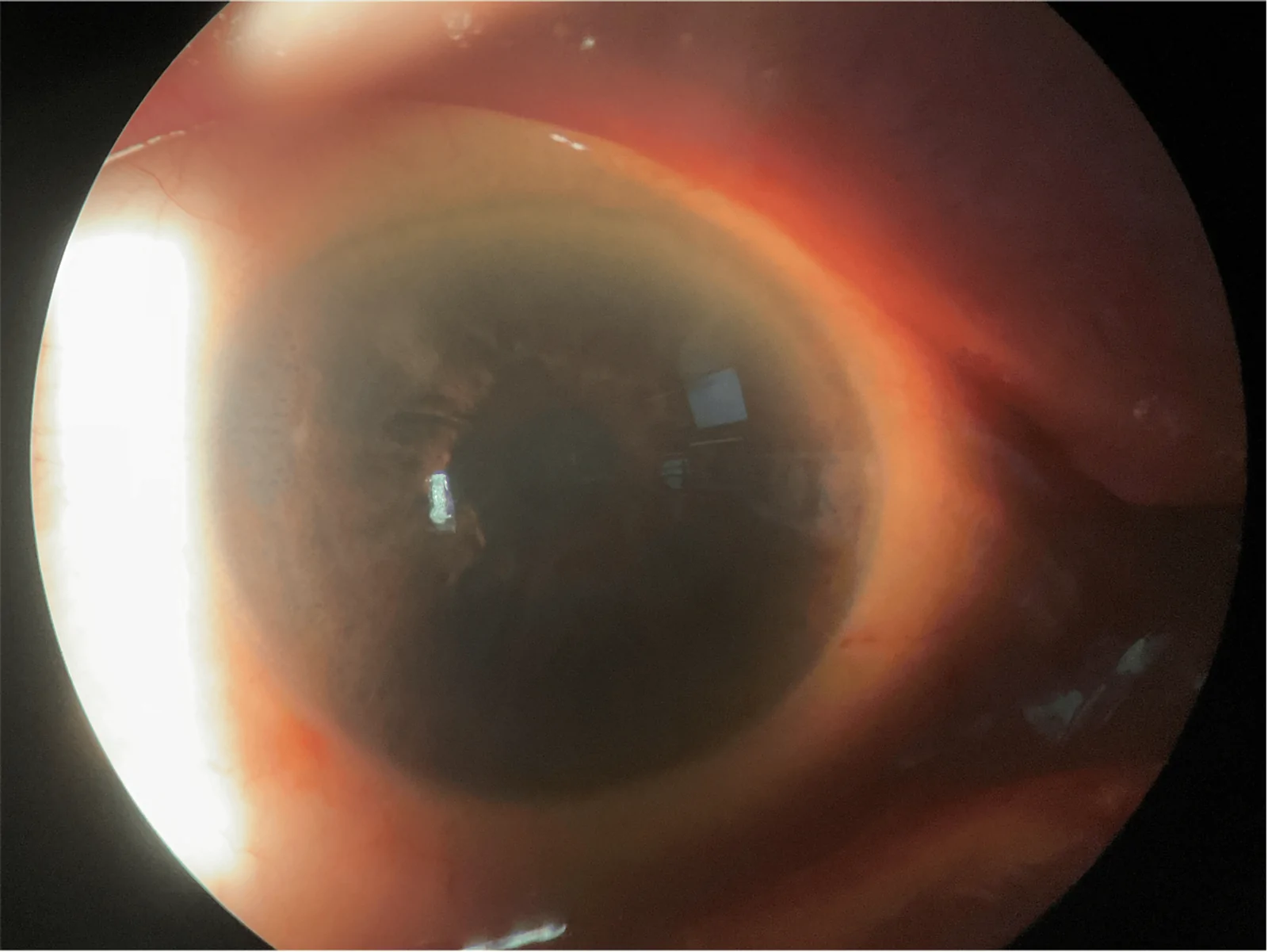
Slit-lamp photograph two months after discontinuation of the PARP inhibitor olaparib. Retroillumination image. The disciform keratitis had resolved. PARP: Poly(adenosine diphosphate-ribose) polymerase.

BCVA in both eyes remained around 1.2 during the observation period.

## Discussion

While herpetic keratitis is an ophthalmic disease, research in recent years has advanced beyond its purely ophthalmological aspects, such as diagnosis and treatment, to explore the relationship between the condition and systemic diseases. It has been reported that patients with diabetes face an increased risk of developing herpetic keratitis, as hyperglycemia can cause damage to the corneal epithelium [8]. In addition to the initial infection, the immune response plays a crucial role in the pathogenesis of herpetic keratitis [5]. Patients infected with the HIV, a prototypical immunodeficiency condition, are reported to experience a higher incidence of severe herpetic keratitis and a greater likelihood of recurrence [9-11]. Diabetes has been shown not only to cause corneal epithelial damage but also to impair immune responses systemically [12]. Consequently, a compromised immune response is considered a potential factor contributing to the development of herpetic keratitis in diabetic patients. These findings reinforce the understanding that the immune response is critical to the pathogenesis of herpetic keratitis [5].

There have been reports of patients developing herpes simplex virus (HSV)-associated endotheliitis while receiving topical interferon (IFN)-α2b therapy for ocular surface squamous cell carcinoma [13]. Cases of disciform keratitis have also been reported in association with monkeypox virus (MPXV) infection, a condition frequently observed in immunocompromised individuals [14]. Although the condition initially resolved under the suspicion of herpetic keratitis, MPXV DNA was subsequently identified as the causative agent via PCR analysis of aqueous humor [14]. Thus, disciform keratitis can develop in various situations where immune suppression may occur. Furthermore, it is important to consider that pathogens not previously suspected of triggering disciform keratitis may also be potential causes of the condition.

We encountered a case involving a patient with a history of ovarian cancer undergoing chemotherapy, in whom disciform keratitis developed, went into remission, and subsequently relapsed. The disciform keratitis resolved during treatment with a PARP inhibitor, a period characterized by systemic immunosuppression, but relapsed upon discontinuation of treatment. This pattern was observed repeatedly. As previously discussed, it is readily conceivable that the systemic immune response plays a crucial role in the pathogenesis of herpetic keratitis, particularly in the development of disciform keratitis, which is driven by immune-mediated mechanisms. However, there have been no reports in clinical practice that compare and analyze fluctuations in a patient's immune status alongside the clinical course of disciform keratitis. The information obtained from this case may provide evidence that systemic immune status contributes to the recurrence of disciform keratitis. However, data on hematological and immunological parameters (such as CBC with leukocyte differential and lymphocyte counts) were not available. Furthermore, this report demonstrated an association in a single case; it does not establish a causal relationship. The assertion that systemic immune status influences the resolution or recurrence of lesions lacks support from actual immunological data, and therefore, the hypothesis has not been empirically verified and has limitations.

Taken altogether, for patients with cancer, it is essential, not only for disciform keratitis but also for determining treatment strategies for any ophthalmic condition, to verify and fully understand the details of their ongoing medical treatment and to correlate these with clinical ocular findings.

## Conclusions

In a patient with a history of ovarian cancer, disciform keratitis resolved during treatment with PARP inhibitors. However, the condition was observed to recur following the discontinuation of chemotherapy. It has been reported that the systemic immune response plays a crucial role in the pathogenesis of herpetic keratitis, particularly in disciform keratitis, which is driven by immune-mediated mechanisms. There have been no previous reports in clinical practice comparing and examining the relationship between changes in a patient's immune status and the course of disciform keratitis; thus, the information obtained from this case may provide valuable, direct evidence that systemic immune status influences the clinical course of disciform keratitis.
